# Gremlin Activates the Smad Pathway Linked to Epithelial Mesenchymal Transdifferentiation in Cultured Tubular Epithelial Cells

**DOI:** 10.1155/2014/802841

**Published:** 2014-05-18

**Authors:** Raquel Rodrigues-Diez, Raúl R. Rodrigues-Diez, Carolina Lavoz, Gisselle Carvajal, Alejandra Droguett, Ana B. Garcia-Redondo, Isabel Rodriguez, Alberto Ortiz, Jesús Egido, Sergio Mezzano, Marta Ruiz-Ortega

**Affiliations:** ^1^Cellular Biology in Renal Diseases Laboratory, IIS-Fundación Jiménez Díaz, Universidad Autónoma de Madrid, Avenida Reyes Católicos 2, 28040 Madrid, Spain; ^2^Division of Nephrology, School of Medicine, Universidad Austral, Valdivia, Chile; ^3^Bone and Mineral Research Unit, Hospital Universitario Central de Asturias, 33006 Oviedo, Spain; ^4^Instituto Reina Sofía de Investigación Nefrológica, 28003 Madrid, Spain; ^5^Division of Dialysis, IIS-Fundación Jiménez Díaz, Universidad Autónoma de Madrid, Avenida Reyes Católicos 2, 28040 Madrid, Spain; ^6^Division of Nephrology and Hypertension, IIS-Fundación Jiménez Díaz, Universidad Autónoma de Madrid, CIBERDEM, Avenida Reyes Católicos 2, 28040 Madrid, Spain

## Abstract

Gremlin is a developmental gene upregulated in human chronic kidney disease and in renal cells in response to transforming growth factor-**β** (TGF-**β**). Epithelial mesenchymal transition (EMT) is one process involved in renal fibrosis. In tubular epithelial cells we have recently described that Gremlin induces EMT and acts as a downstream TGF-**β** mediator. Our aim was to investigate whether Gremlin participates in EMT by the regulation of the Smad pathway. Stimulation of human tubular epithelial cells (HK2) with Gremlin caused an early activation of the Smad signaling pathway (Smad 2/3 phosphorylation, nuclear translocation, and Smad-dependent gene transcription). The blockade of TGF-**β**, by a neutralizing antibody against active TGF-**β**, did not modify Gremlin-induced early Smad activation. These data show that Gremlin directly, by a TGF-**β** independent process, activates the Smad pathway. In tubular epithelial cells long-term incubation with Gremlin increased TGF-**β** production and caused a sustained Smad activation and a phenotype conversion into myofibroblasts-like cells. Smad 7 overexpression, which blocks Smad 2/3 activation, diminished EMT changes observed in Gremlin-transfected tubuloepithelial cells. TGF-**β** neutralization also diminished Gremlin-induced EMT changes. In conclusion, we propose that Gremlin could participate in renal fibrosis by inducing EMT in tubular epithelial cells through activation of Smad pathway and induction of TGF-**β**.

## 1. Introduction

Many embryological expressed genes regulate morphogenesis and then become quiescent in the normal adult kidney. Recent studies have shown that some developmental genes are reactivated in the adult diseased kidneys [1]. The reemergence of these genes appears to be linked to tissue repair, but when an imprecise interaction of developmental and inflammatory signals occurs, complete healing is not achieved. Instead, there is an excessive production of matrix proteins leading to a scar formation. Gremlin was identified as one of the developmental genes induced in cultured human mesangial cells exposed to high glucose, initially known as induced in high glucose-2 (IHG-2) and also called downregulated by mos (Drm) [2]. Gremlin is a member of cysteine knot superfamily [3] that includes transforming growth factor-*β* (TGF-*β*) proteins and acts as a bone morphogenetic protein (BMP) antagonist [4]. Analysis of the predicted amino acid sequence indicated the presence of several significant features, including potential nuclear localization signals near the C-terminus, potential N-linked glycosylation sites, and multiple potential sites for phosphorylation. The signalling peptide and a predicted glycosylation site have been identified. Gremlin is a glycosylated, phosphorylated, secreted protein present both on the external cell surface and within the ER-Golgi compartments [3]. In many human renal diseases induction of Gremlin has been described [5–8]. Several experimental studies have shown that Gremlin participates in renal damage [9, 10]. Therefore, some authors have suggested that Gremlin could be considered as a mediator of renal injury.

Chronic progressive fibrosis of the kidney remains an unresolved challenge. Irrespective of the underlying cause, chronic kidney disease is linked to the development of tubulointerstitial fibrosis, characterized by accumulation of extracellular matrix (ECM). The mechanisms of renal fibrosis are complex and our therapeutic armamentarium is limited. The key cellular mediator of fibrosis is the myofibroblast. There are different sources of myofibroblasts, including activation of tissue fibroblasts and migration of circulating mesenchymal progenitors or cell transitions, including epithelial-mesenchymal transition (EMT) or endothelial mesenchymal transition [11, 12]. The investigation of the mechanisms involved in renal fibrosis and the identification of novel mediators with potential therapeutic application is an important open question in chronic kidney disease.

TGF-*β*1, signaling mainly through Smad proteins, is a key player in fibrosis and EMT [13–17]. Because of its pleiotropic actions, TGF-*β* blockade is not an ideal therapeutic tool; therefore, novel targets are needed. Among them, Gremlin may be an interesting candidate in progressive renal diseases. Recent* in vitro* studies developed by our group have shown that Gremlin gene silencing inhibited TGF-*β*-mediated matrix production and EMT [18]. However, the involvement of TGF-*β* in Gremlin responses has not been investigated. We have also reported the presence of Gremlin in glomerular crescents of human pauci-immune glomerulonephritis and in the tubulointerstitium of chronic allograft nephropathy. In these human diseases Gremlin correlated with the degree of tubulointerstitial fibrosis and was associated with TGF-*β*1 overexpression and Smad pathway activation [7, 8]. These studies suggest that Gremlin may activate the Smad pathway; therefore, the aim of this work was to evaluate whether Gremlin could directly activate the Smad pathway in tubular epithelial cells, evaluating whether this activation is linked to Gremlin-induced EMT, the main fibrotic effect observed in response to Gremlin stimulation in these cells [18].

## 2. Materials and Methods 

### 2.1. Cell Cultures

Human renal proximal tubuloepithelial cells (HK2 cell line, ATCC CRL-2190) were grown in RPMI with 10% fetal bovine serum (FBS), 1% nonessential amino acids, 100 U/mL penicillin, and 100 *μ*g/mL streptomycin, insulin transferrin selenite (ITS) (5 *μ*g/mL), and hydrocortisone (36 ng/mL) in 5% CO_2_ at 37°C. At 60–70% of confluence, cells were growth-arrested in serum-free medium for 24 hours before the experiments. Then, cells were stimulated for different times with recombinant Gremlin (50 ng/mL) (R&D) or human recombinant TGF-*β*1 (1 ng/mL, Peprotech). Cell culture reagents were obtained from Life Technologies Inc. TGF-*β* was targeted by a pan-specific polyclonal anti-TGF-*β* neutralizing antibody, which recognizes bovine, mouse, and human TGF-*β*1 and *β*2 isoforms (1 *μ*g/mL) (R&D).

### 2.2. Transfection, cDNA Constructs, and Promoter Studies

HK2 cells were transiently transfected for 24–48 hours with FuGENE (Roche), pCDNA3-Gremlin-myc-IRES2-eGFP plasmid (GREM-GFP) and/or pCDNA-FLAG-Smad7 expression vector (kindly donated by Dr. Massagué, Memorial Sloan-Keternig Cancer Center, USA) or empty vector (pCDNA). The GREM-GFP was generated as follows: GREM1 cDNA was purchased from the Mammalian Gene NIH Collection (Bethesda, Maryland, USA). We added a c-myc tag to the 3′ portion of GREM1 using PCR with the forward primer 5′AGTGCGGCGGCTGAGGACCC GCCGCACTGACAT-3′ and the reverse primer 5′-ATAGCCGCCGCTTACAGATCCTCTTCTGAGATGAGTTTTTGTTCATCCAAATCGATGGATATGC-3′. We also inserted an e-GFP sequence downstream of human Gremlin as follows. The IRES-eGFP sequence was obtained by PCR using a pIRES2-EGFP plasmid (Clontech Mountain View, CA, USA) as the template with the following primers: IRES-eGFP-F (5′-TACATTAATGGGCCCGGGATCCGCCCCTC-3′) and IRES-eGFP-R (5′-GGCCATATGCGCCTTAAGATACATTGATG-3′). The GREM1-c-myc and IRES-eGFP fragments were independently cloned into a pGEMT-Easy vector and then sequenced (Macrogen, Seoul, Korea) to confirm the modifications and absence of additional mutations. Next, both the GREM1-c-myc and IRES-eGFP fragments were subcloned into a modified pCDNA3 vector using the* Eco*RI and* Not*I restriction sites, respectively. In Gremlin-transfected cells, Gremlin production was confirmed by immunofluorescence (not shown).

To demonstrate Smad 7 transfection efficacy an anti-FLAG antibody was used (not shown). Smad-dependent promoter activation was evaluated by transfection of Smad/luc (kindly donated by Dr. Volgestein, Baltimore, USA) and TK-renilla as internal control, as described [19].

### 2.3. Protein Studies

Total cellular protein extracts (10–50 *μ*g/lane) obtained in lysis buffer [50 mM Tris-HCl, pH 7.4, 150 mM NaCl, 2 mM EDTA, 2 mM EGTA, 0.2% Triton X-100, 0.3% NP40, 100 *μ*M phenylmethylsulphonylfluoride, 1 mM dithiothreitol, 100 *μ*M Na_3_VO_4_, and 1 mM protease-inhibitor cocktail (Sigma)] were separated on 8–12% polyacrylamide-SDS gels under reducing conditions. Samples were then transferred onto nitrocellulose membranes (Bio-Rad, Hercules, CA), blocked with 5% nonfat dry milk, in 50 mM Tris-HCl, pH 7.5, 150 mM NaCl with 0.05% Tween-20, and incubated overnight at 4°C with the primary antibodies and subsequently incubated with peroxidase-conjugated IgG (Amersham), and developed by ECL chemiluminescence (GE Healthcare, Buckinghamshire, UK).

Immunocytochemistry studies were performed in cells growing on coverslips. After the experiments, cells were fixed in Merckofix (Merck) and permeabilized with 0.2% Triton-X100 for 10 min (except for E-cadherin staining). After blocking with 4% BSA and 8% serum for 1 hour, samples were incubated with primary antibodies overnight at 4°C, and then 1 hour at room temperature with fluorescein isothiocyanate (FITC) [1/200] or AlexaFluor 633 [1/300] conjugated antibodies (Amersham). Nuclei were stained with 1 *μ*g/mL propidium iodide (PI) or 4′,6-Diamidino-2-phenyindole dilactate (DAPI) (Sigma-Aldrich), as control of equal cell density. Absence of primary antibody was used as negative control. Samples were mounted in Mowiol 40–88 (Sigma-Aldrich) and examined by a Leica DM-IRB confocal microscope.

The antibodies employed were: p-Smad 3 (Abcam) (WB: 1/1000), Smad 2 and Smad 4 (Sta. Cruz) (IF: 1/300), Smad3 (Sta Cruz) (IF: 1/300, WB: 1/1000), Vimentin (BD Pharmingen) (IF: 1/200; WB: 1/1000), E-cadherin (R&D) (IF: 1/200, WB: 1/1000), Slug (Cell signaling) (WB: 1/1000), pan-Cytokeratin, and *α* -SMA (Sigma Aldrich) (IF: 1/200).

TGF-*β*1 protein was measured in the cell-conditioned medium using a commercial enzyme-linked immunoassay (ELISA) (BD Sciences, San Diego, USA) following the manufacturer's instructions. TGF-*β*1 levels were quantified by comparison with a standard curve using increasing concentrations of human TGF-*β*1. Protein content was determined by the BCA method (Pierce).

### 2.4. Gene Expression

Total RNA was isolated from cells with Trizol (Invitrogen) according to the manufacturer's protocol. cDNA was synthesized from 2 *μ*g of total RNA with random hexamer primers using the High capacity cDNA Archive Kit (Applied). Real-time PCR was performed using human FAM TaqMan MGB probes designed by assay-on-demand gene expression products (Applied): TGF-*β*1: Hs99999918_m1; connective tissue growth factor (CTGF): Hs00170014_m1: plasminogen activator inhibitor 1 (PAI1): Hs 00167155_m1. Data weres normalized to 18S eukaryotic ribosomal RNA: 4210893E (VIC). The mRNA copy numbers were calculated for each sample by the instrument software using Ct value (“arithmetic fit point analysis for the lightcycler”). Results were expressed in copy numbers and calculated relative to unstimulated cells after normalization against 18S.

### 2.5. Statistical Analysis

Results throughout the text are expressed as mean ± SEM. Differences between agonist-treated groups and controls were assessed by one-way analysis of variance, followed by post hoc Bonferroni or Dunnett test or Mann-Whitney test, as appropriate. *P* < 0.05 was considered significant. Statistical analysis was conducted using the SPSS statistical software, version 11.0 (SPSS).

## 3. Results

### 3.1. Gremlin Activates Smad Pathway in Human Cultured Tubuloepithelial Cells

**Figure 1:**
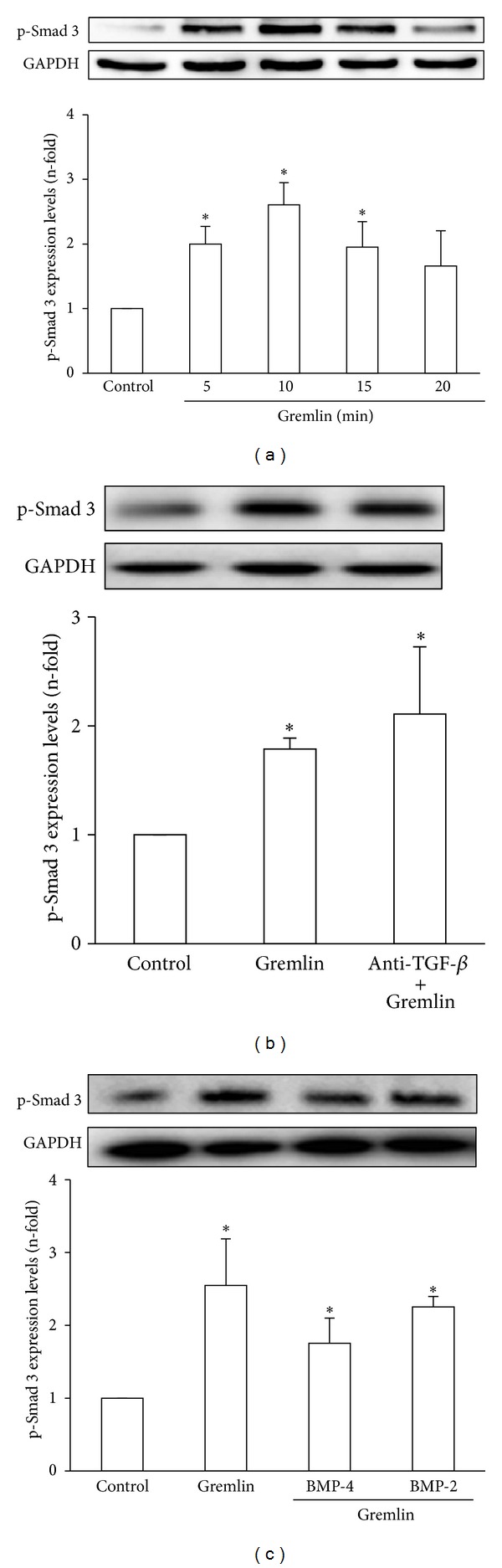
*(a) Stimulation with Gremlin rapidly increased Smad 3 phosphorylation in cultured human tubuloepithelial cells*. HK2 cells were stimulated with Gremlin (50 ng/mL) for different times.* (b) Early Smad 3 phosphorylation induced by stimulation with Gremlin was not mediated by TGF-*β**. TGF-*β* was blocked or not (control) by pretreatment of cells for 1 hour with an anti-TGF-*β* neutralizing antibody and then treated with Gremlin for 10 minutes. (c) In some points, HK2 cells were preincubated with BMP-2 or BMP-4 and then treated with Gremlin for 10 minutes. Total proteins were isolated and protein levels were evaluated by western blot. GAPDH or Smad 3 were used as loading controls. Figures show a representative western blot of phosphorylated levels of Smad 3 and data are expressed as n-fold over control (considered as 1), as the mean ± SEM of 3-4 independent experiments. *P < 0.05 versus control.

Receptor mediated activation of Smad proteins (R-Smads 2 and 3) occurs by direct C-terminal phosphorylation. Smad 2/3 then form complexes with Smad 4 and translocate into the nucleus, where they associate and cooperate with DNA binding transcription factors to activate or repress target gene transcription [17]. In cultured HK2 cells, stimulation with recombinant Gremlin increased phosphorylation levels of Smad 3 as early as 5 minutes, and it was maintained until 15 minutes (Figure 1(a)).

Although Smad is the main signaling mechanism of TGF-*β*, several factors involved in renal damage, such as angiotensin II, can directly activate the Smad pathway, independent of endogenous TGF-*β* [17]. Therefore, to evaluate whether early Smad activation caused by Gremlin was mediated or not by TGF-*β*, cells were preincubated with a neutralizing antibody against active TGF-*β*. Gremlin-induced Smad activation (evaluated as p-Smad 3 levels) was not modified in the presence of the TGF-*β* antibody (Figure 1(b)). Similar lack of response was found in the presence of decorin (a proteoglycan that neutralizes active TGF-*β*, not shown). These data indicates that Gremlin directly activates the Smad pathway.

Some actions of Gremlin are due to its effect as BMP antagonist [4]. To determine the contribution of BMPs in Gremlin-induced Smad activation, HK2 cells were preincubated with BMP-2 or BMP-4 and then stimulated with Gremlin during 10 minutes. Phosphorylation of Smad 3 was not modified in the presence of any of these BMPs (Figure 1(c)), suggesting that Gremlin-induced Smad activation is independent of BMPs in tubular epithelial cells.

**Figure 2:**
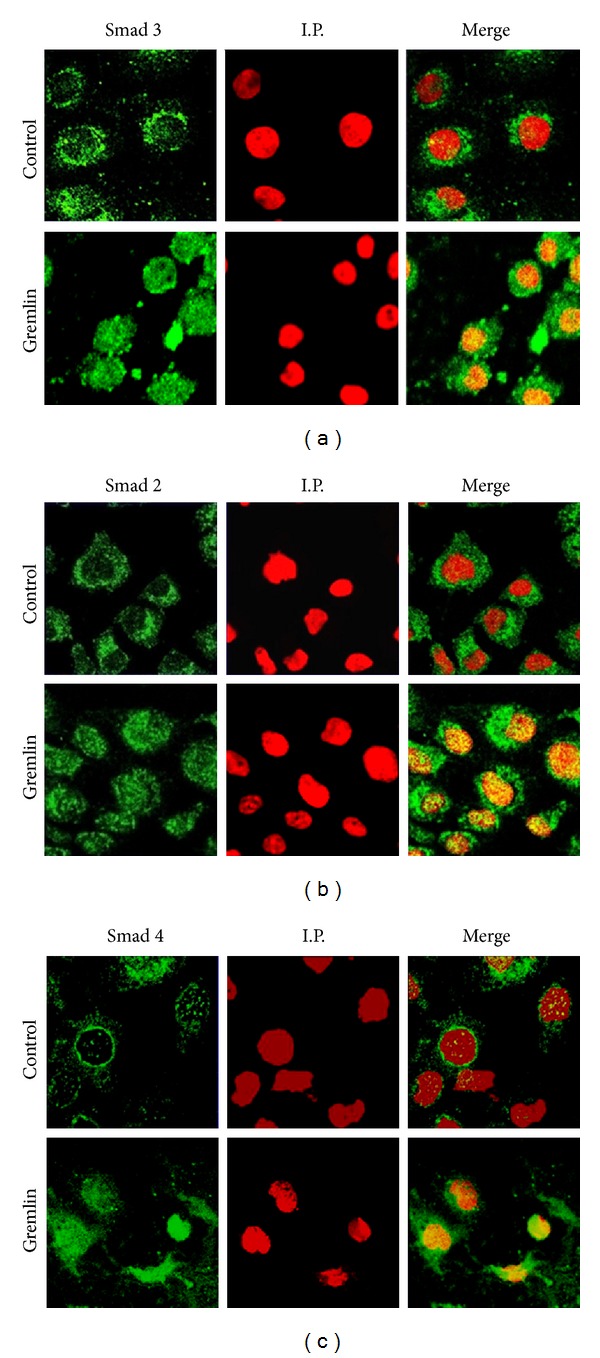
*Stimulation with Gremlin induces a rapid activation of the Smad pathway in cultured human tubuloepithelial cells*. HK2 cells were stimulated with Gremlin (50 ng/mL) for 15 minutes. The localization of R-Smad 3 (a) and 2 (b) and Smad 4 (c) was evaluated by confocal microscopy with FITC-secondary antibodies (green staining). Nuclei were stained with propidium iodide (I.P.) (red). In the merge, the yellow staining indicates the nuclear localization of Smad proteins. The results are representative of 3 independent confocal microscopy experiments.

By confocal microscopy, we have confirmed that Gremlin rapidly increased Smad 3 translocation to the nucleus; the latter demonstrated by the yellow nuclear staining observed in the merge of Figure 2, while in untreated cells, the nuclei are red. Gremlin also increased nuclear localization of Smad 2 and Smad 4, observed at 15 minutes (Figure 2).

**Figure 3:**
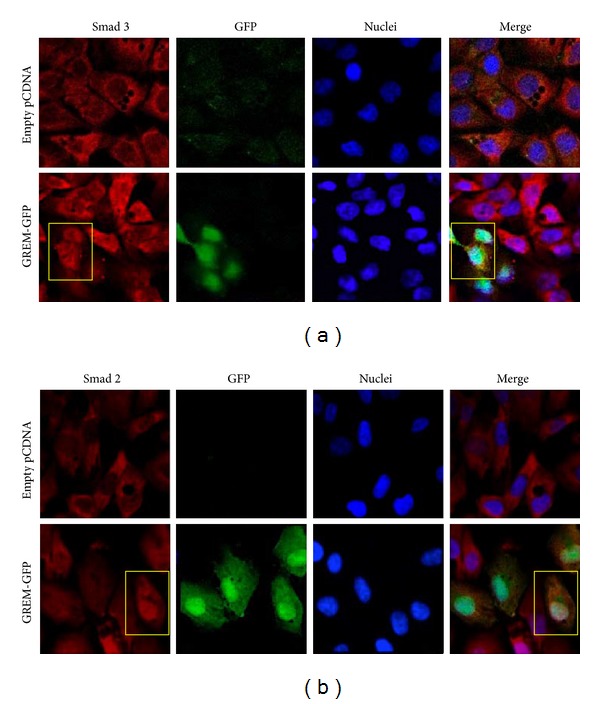
*Gremlin overexpression causes a sustained Smad activation in cultured human tubuloepithelial cells*. HK2 cells were transiently transfected with a Gremlin expression vector (GREM-GFP; green) or empty vector for 24 hours. The levels and localization of R-Smad 3 (a) and R-Smad 2 (b) were evaluated by confocal microscopy with Alexa-633 secondary IgG (red). Nuclei were stained using 4′,6-diamino-2-phenylindole dihydrochloride (DAPI; blue). In Gremlin-transfected cells (green staining by GFP), the Smad 2 and Smad 3 were found in the nuclei (white staining in the merge). Figures show representative images out of 3 independent observations.

To further confirm that Gremlin activates the Smad pathway, cells were transfected with a Gremlin expression vector (GREM-GFP) for 24 hours. By confocal microscopy we observed that in positive Gremlin-transfected cells (GFP-green staining) there was a nuclear immunostaining showing the translocation of Smad 3 and Smad 2 (characteristic of Smad activation), compared to cells transfected with empty vector (Figures 3(a) and 3(b)). In the merge of Figure 3, a Gremlin expressing-cell marked by a yellow rectangle presented a positive nuclear staining, corresponding to the presence of Smad 3 or 2. In contrast, in cells transfected with empty vector, there are no nuclear Smad 2 and Smad 3 immunostaining (all cells present blue nuclei), as observed in some nontransfected cells.

**Figure 4:**
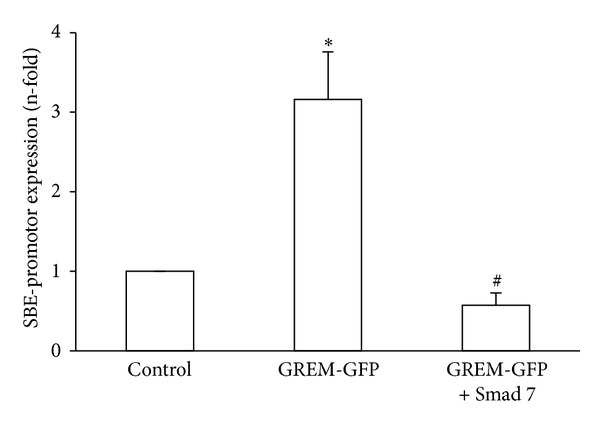
*Gremlin overexpression induces Smad-dependent gene transcription*. HK2 cells were transfected with GREM-GFP or empty vector, Smad/luc promoter, and TK-renilla for 24 hours. In some points, cells were cotransfected with Smad 7. Then, luciferase/renilla activity was measured. Data are expressed as increase in Smad binding element (SBE) promoter-luciferase dependent expression. Data are expressed as n-fold over control (considered as 1), as the mean ± SEM of 5 experiments. *P < 0.05 versus control. ^#^ *P* < 0.05 versus Gremlin.

To investigate whether Gremlin regulates Smad-mediated gene expression, cells were cotransfected with a Gremlin expression vector (GREM-GFP) and a luciferase Smad reporter plasmid. Gremlin transfected cells expressed higher Smad-dependent luciferase activity (SBE) than control cells (Figure 4). To demonstrate further the involvement of Smad pathway in Gremlin-induced responses, a Smad 7 expression vector, that inhibits Smad-mediated transcriptional effects by interfering with receptor-mediated activation of R-Smad, was used [17, 19]. In HK2 cells cotransfected with GREM-GFP and Smad 7 expression vectors, the Smad-mediated luciferase activity was significantly lower than cells transfected with GREM-GFP alone, showing the specific Smad 7 blockade of Gremlin-mediated Smad activation (Figure 4).

### 3.2. Gremlin-Induced EMT Is Mediated by Smad Activation

**Figure 5:**
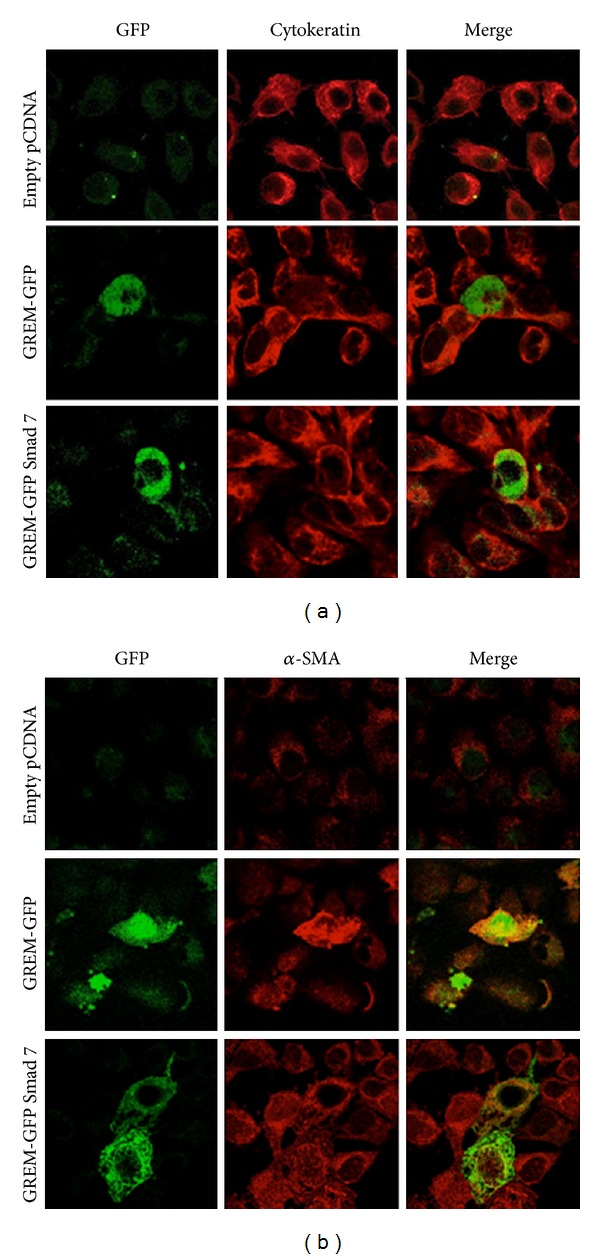
*Gremlin-induced EMT via the Smad pathway*. HK2 cells were transiently transfected with empty, Gremlin (GREM-GFP) alone or cotransfected with Smad 7 expression vectors. EMT markers were evaluated after 48 hours. Gremlin transfected cells express GFP (green staining). Confocal microscopy analysis of cytokeratin and *α*-SMA immunofluorescence was performed using specific primary antibodies and Alexa-633 secondary IgG (red staining). Representative image out of 3 experiments.

We have previously demonstrated that in tubular epithelial cells long-term stimulation with recombinant Gremlin induced EMT [18]. Now, we have observed that transfection of HK2 cells with GREM-GFP induced EMT-related phenotypic changes observed by confocal microscopy after 48 hours (Figure 5). Cells transfected with empty-vector showed epithelial morphology, including the presence of epithelial markers, such as cytokeratin (red staining), and there is no positive staining for mesenchymal marker *α*-SMA (Figure 5). In contrast, overexpression of Gremlin caused changes in morphology to fibroblast-like shape and induction of *α*-SMA (see the GREM-GFP positive cell that presents yellow staining and elongated shape). Moreover, in Gremlin expressing cells cytokeratin staining was markedly diminished (absence of red staining in an area with several GREM-GFP positive green cells). The blockade of Smad activation, by cotransfection with Gremlin and Smad 7, diminished these EMT changes (Figure 5), as shown by restoration of the cytokeratin immunostaining and the epithelial morphology and diminution of *α*-SMA as observed in the green positive cell. These data suggest that Gremlin regulates EMT through the Smad pathway.

### 3.3. Role of Endogenous TGF-*β* on Gremlin-Induced EMT

**Figure 6:**
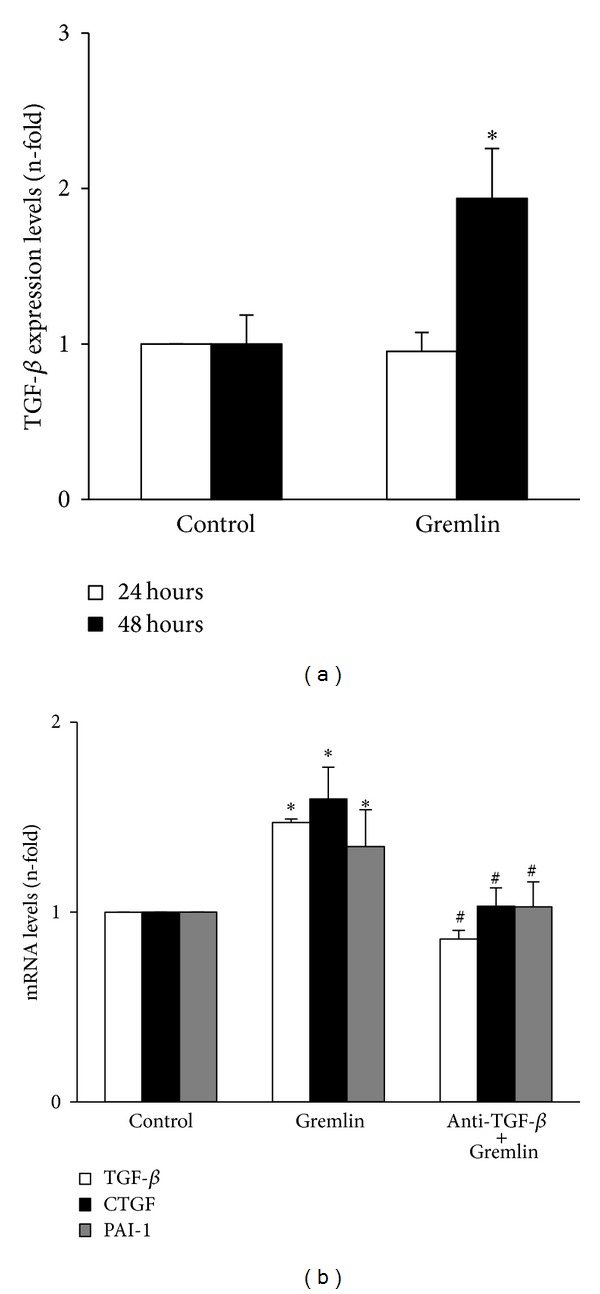
*(a) Gremlin increased TGF-*β* production*. HK2 cells were stimulated with Gremlin (50 ng/mL) for 24 and 48 hours in serum-free medium. TGF-*β*1 protein levels were measured in the cell-conditioned medium using a specific ELISA. Data are expressed as mean ± SEM of 6 independent experiments. *P < 0.05 versus control.* (b) The late increase in gene expression of profibrotic factors caused by Gremlin is mediated by endogenous TGF-*β* production*. HK2 cells were stimulated with Gremlin (50 ng/mL) for 24 hours in serum-free medium. TGF-*β* was blocked or not (control) by pretreatment of cells for 1 hour with an anti-TGF-*β* neutralizing antibody. Total cell RNA was isolated to assess mRNA levels by real-time PCR. Data are expressed as n-fold over control (considered as 1), as the mean ± SEM of 3 experiments. *P < 0.05 versus control. ^#^ *P* < 0.05 versus Gremlin.

**Figure 7:**
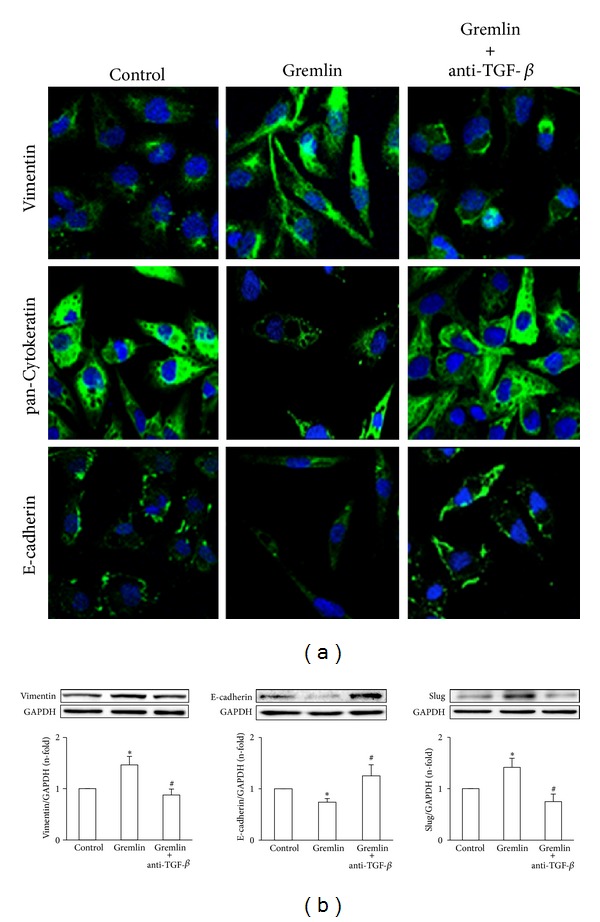
*TGF-*β* is a mediator of EMT-related changes following stimulation with Gremlin*. HK2 cells were stimulated with Gremlin (50 ng/mL) for 48 hours in serum-free medium. TGF-*β* was blocked or not (control) by pretreatment of cells for 1 hour with an anti-TGF-*β* neutralizing antibody. (a) EMT changes were evaluated by confocal microscopy. E-cadherin, pan-Cytokeratin, and Vimentin were studied by indirect immunofluorescence using FITC-secondary IgG (green) and confocal microscopy. Nuclei are shown in blue. Figure shows a representative image out of 3 independent observations. (b)Total proteins were isolated and Vimentin, E-cadherin, and Slug levels were analyzed by western blot. Data are expressed as n-fold over control (considered as 1), as the mean ± SEM of 3 experiments. *P < 0.05 versus control. ^#^ *P* < 0.05 versus Gremlin.

Previously, we have reported that Gremlin acts as a downstream mediator of TGF-*β*-induced fibrosis in cultured renal cells and incubation with Gremlin for 24 hours induced a significant upregulation of TGF-*β*1 mRNA levels in cultured tubuloepithelial cells [18]. We have further investigated the relation between Gremlin/TGF-*β*, evaluating whether Gremlin could regulate TGF-*β*1 synthesis. In HK2 cells, active TGF-*β*1 protein increased in the supernatants of Gremlin-stimulated cells after 48 hours, but not at 24 hours (Figure 6(a)), suggesting that some of the profibrotic actions of Gremlin could be mediated by endogenous TGF-*β*1 synthesis. Therefore, we blocked TGF-*β* before HK-2 stimulation with Gremlin by adding a neutralizing antibody against active TGF-*β*, which is able to block angiotensin II-induced ECM production and EMT [19, 20]. TGF-*β* neutralization inhibited Gremlin-induced gene upregulation of profibrotic factors observed after 24 hours, including TGF-*β*, CTGF, and PAI-1 (Figure 6(b)). Moreover, TGF-*β* blockade antagonized several EMT-related changes induced by Gremlin after 48 hours, as shown by immunofluorescence (Figure 7(a)). We also observed by western blot that TGF-*β* neutralization diminished Vimentin and Slug induction caused by Gremlin and restored E-cadherin levels decreased by Gremlin (Figure 7(b)). These data suggest that TGF-*β* is a mediator of long-term responses of Gremlin in tubuloepithelial cells, including regulation of profibrotic factors and EMT changes.

## 4. Discussion 

Our* in vitro* studies in cultured tubuloepithelial cells show that Gremlin directly activates the Smad pathway and participates in the EMT process, via Smad signalling. These data suggest that Gremlin could be a mediator of renal fibrosis.

**Figure 8:**
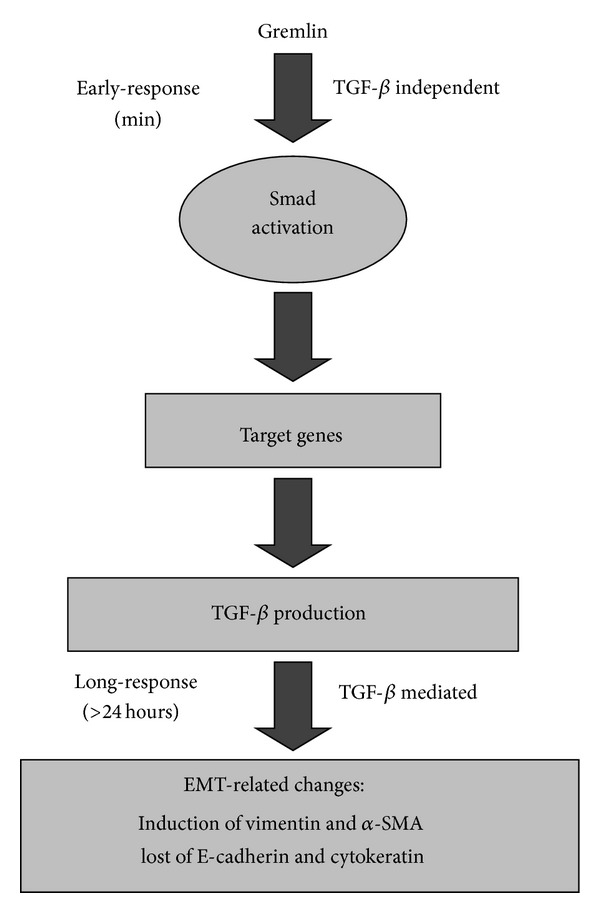
*Dual effects of Gremlin on Smad activation*. Gremlin induces an early (minutes) and direct, TGF-*β*-independent, Smad pathway activation. After 24 hours Gremlin increased several profibrotic genes, including TGF-*β*, and after 48 hours increased TGF-*β* production and induced EMT features. These long-term Gremlin-induced profibrotic events require autocrine TGF-*β*.

Our study reveals that in cultured human tubuloepithelial cells Gremlin induces a rapid activation of the Smad pathway (observed after 5 min of stimulation) characterized by increased phosphorylation of the receptor-Smad (R-Smad), Smad 3, a critical downstream mediator of fibrosis [17], and Smad 2 proteins. Once R-Smad is phosphorylated it dimerises with Smad 4 and then shuttles to the nucleus to regulate gene expression. By confocal microscopy, we have found that Gremlin caused a rapid translocation to the nucleus of R-Smad/Smad 4 proteins. In several cells types Gremlin-induced TGF-*β* production [18, 21], as we have observed here after 48 hours of incubation. However, Gremlin-induced early Smad activation is independent of endogenous TGF-*β*, as we have demonstrated using TGF-*β* blockers (Figure 8). Other important profibrotic factors, such as angiotensin II, also activates the Smad pathway, rapidly and independent of endogenous TGF-*β* [17].

Previous studies in tubular epithelial cells have shown that the Smad route regulates EMT induced by key factors involved in renal fibrosis, such as TGF-*β* and angiotensin II [20]. The activation of Smad pathway has been described in experimental renal fibrotic diseases, including glomerulosclerosis, tubulointerstitial fibrosis, hypertensive-induced renal damage, and diabetic nephropathy [20–26], as well as in renal tumor progression [27]. In angiotensin II-induced renal damage, renal activation of the Smad pathway was associated to EMT changes [20]. Moreover, Smad 7 overexpression ameliorates renal damage and fibrosis caused by unilateral ureteral obstruction, angiotensin II, and diabetes [22–24, 28]. We have observed that in tubuloepithelial cells Smad 7 overexpression blocked Gremlin-induced EMT changes. The involvement of Smad pathway in Gremlin-mediated fibrosis has been also described in other cell types* in vitro*. In optic nerve head astrocytes and lamina cribrosa cells recombinant Gremlin stimulates ECM production through the activation of TGF-*β* receptor and Smad 3 phosphorylation, suggesting a role for Gremlin in glaucoma [29]. In healthy dermal fibroblasts IL-6 mediated induction of collagen is dependent on Gremlin production and activation of TGF-*β*/Smad signalling [30]. Besides the regulation of renal EMT and fibrosis, Gremlin/Smad pathway could also be involved in the onset of proteinuria by modulating podocyte injury and changing the distribution of nephrin and synaptopodin [21].

Recent evidences suggest that Gremlin could be an important promoter of fibrosis in different pathologies, including liver fibrosis, lung diseases, particularly pulmonary hypertension and idiopathic pulmonary fibrosis, and myocardial fibrosis [31–35]. In several human renal diseases Gremlin overexpression was found, mainly in areas of tubule interstitial fibrosis [5–8]. Experimental studies in mice have shown that Gremlin blockade diminished renal fibrosis, as observed in streptozotocin-induced diabetes in knockout mice heterozygous for grem1 [9] and by Gremlin gene silencing [10]. Recent studies have demonstrated direct fibrogenic effect of Gremlin in renal cells. In mesangial cells Gremlin increased cell proliferation and ECM accumulation, via ERK [36]. In renal fibroblasts Gremlin increased ECM production [18], including type I collagen. In tubular epithelial cells Gremlin upregulates profibrotic genes, such as TGF-*β* and CTGF, and caused EMT changes [18]. Gremlin also induces EMT in airway epithelial cells [37] and in cancer cells [38]. Although the contribution of EMT to renal fibrosis is a matter of intense debate [39, 40], the lost of epithelial properties of the tubular epithelial cells, including permeability and polarity, may result in decreased viability and contribute to renal injury [40, 41]. Therefore, EMT-related changes are an initial step in renal damage and an important potential therapeutic target. Our data demonstrate that Gremlin via Smad pathway regulates EMT, showing a novel mechanism of Gremlin action in renal cells.

TGF-*β* is known as the major promoter of EMT during embryogenesis, cancer, and fibrosis [13–17].In a mesothelioma cell line Gremlin-silencing inhibited cell proliferation, associated with downregulation of the transcription factor slug as well as mesenchymal proteins linked to cancer EMT [38]. We have recently demonstrated that Gremlin gene silencing blocked TGF-*β*-induced EMT in tubular epithelial cells [18]. Now, we have observed that Gremlin increased TGF-*β* production at 48 hours, and this endogenous autocrine TGF-*β* acts as a downstream mediator of Gremlin-induced profibrotic and EMT related factors in cultured human tubuloepithelial cells (Figure 8). All these findings reveal the complex relationship between Gremlin and TGF-*β* in the kidney, disclosing a positive feedback loop connection between them in promoting EMT and fibrosis.

Gremlin exerts a potent inhibitory action via binding to and forming heterodimers with BMP-2, BMP-4, and BMP-7. The binding of Gremlin to selective BMPs prevents ligand-receptor interaction and subsequent downstream signalling. Gremlin acting as a BMPs antagonist plays a critical role during the process of nephrogenesis [4]. BMP-7 is the antagonist of TGF-*β*1 signalling and has been found to inhibit TGF-*β*1-induced renal fibrosis by reversing EMT process [42, 43]. In experimental lungs and pulmonary fibrosis upregulation of Gremlin was associated with downregulation of BMP signalling [31, 32]. Gremlin overexpression has been found to inhibit BMP-4 thus leading to enhance TGF-*β* signalling and ECM deposition in primary open angle glaucoma [44]. However, BMP-independent mechanisms may mediate some actions of Gremlin. Exogenous Gremlin may bind to and act directly on endothelial cells to modulate angiogenesis including endothelial cell migration [45, 46]. We have found that BMPs did no inhibit Gremlin-induced early Smad 3 activation. Thus a receptor-mediated mechanism of action may exist for Gremlin. Therefore, future studies investigating the receptor involved in Gremlin responses in renal cells are needed.

## 5. Conclusion

Chronic progressive fibrosis of the kidney remains an unsolved challenge. The investigation of the mediators and mechanisms involved in renal fibrosis could lead to better diagnostic tools and novel therapeutics approaches. Many studies have shown that renal expression of Gremlin is induced in diabetic nephropathy and in other progressive renal diseases, associated with tubulointerstitial fibrosis and Smad activation [5–8]. We show here that Gremlin activates the Smad signaling pathway and induces TGF-*β* and other related factors involved in EMT and fibrotic events in renal cells. All these data suggest that Gremlin could be a potential novel molecular antifibrotic target and biomarker useful for prognostication, disease monitoring, and therapy.
